# Insight into the structure, physiological function, and role in cancer of m6A readers—YTH domain-containing proteins

**DOI:** 10.1038/s41420-022-00947-0

**Published:** 2022-03-28

**Authors:** Jingyu Liao, Yi Wei, Junnan Liang, Jingyuan Wen, Xiaoping Chen, Bixiang Zhang, Liang Chu

**Affiliations:** Hepatic Surgery Center, Tongji Hospital, Tongji Medical College, Huazhong University of Science and Technology, Wuhan, China

**Keywords:** Cancer therapy, Cancer metabolism, Cancer microenvironment

## Abstract

YT521-B homology (YTH) domain-containing proteins (YTHDF1-3, YTHDC1-2) are the most crucial part of N6-methyladenosine (m6A) readers and play a regulatory role in almost all stages of methylated RNA metabolism and the progression of various cancers. Since m6A is identified as an essential post-transcriptional type, YTH domain-containing proteins have played a key role in the m6A sites of RNA. Hence, it is of great significance to study the interaction between YTH family proteins and m6A-modified RNA metabolism and tumor. In this review, their basic structure and physical functions in RNA transcription, splicing, exporting, stability, and degradation as well as protein translation are introduced. Then we discussed the expression regulation of YTH domain-containing proteins in cancers. Furthermore, we introduced the role of the YTH family in cancer biology and systematically demonstrated their functions in various aspects of tumorigenesis and development. To provide a more institute understanding of the role of YTH family proteins in cancers, we summarized their functions and specific mechanisms in various cancer types and presented their involvement in cancer-related signaling pathways.

## Facts

- YTH domain-containing proteins are the most important m6A readers that function on methylated RNA metabolism.
- YTH domain-containing proteins are directly involved in the development and progression of many tumors.
- YTH domain-containing proteins are upregulated in many cancers, but the precise molecular function in a particular cancer may not be complete.

## Open questions

- What are the specific mechanisms of YTH domain-containing proteins over m6A-modified RNA metabolism?
- What is the exact molecular function of YTH domain-containing proteins in various cancers?
- How do the YTH proteins function as the tumor suppressor or promoter during cancer progression?
- Is there any way to block the function of YTH readers as an effective therapy of cancer development?

## Introduction

Since the first discovery of methylnucleosides in mRNA in 1974 [1], the development of methylated RNA m6A immunoprecipitation sequencing (MeRIP-seq) technology has triggered an upsurge in epigenetic modification study, especially in the m6A epigenetic domain. Of the post-transcriptional modifications of cellular RNA, N6-methyladenosine (m6A) is the most common type of RNA modification in eukaryote RNAs, including transfer RNA (tRNA), ribosomal RNA (rRNA), long noncoding RNA (lncRNA), small nuclear RNA (snRNA) and viral RNA. M6A showed consensus motif RRACH (R = G or A; H = A, C, or U) on more than 25% of human transcripts. More than 70% of m6A sites are located near coding sequence (CDS) and 3′ untranslated region (UTR), and about 20% are located near intron region and 5′UTR [2, 3].

The formation of m6A is caused by m6A methyltransferases (m6A writers). The elimination is done by demethylases (m6A erasers), while recognition is done by m6A-binding proteins (m6A readers). M6A writers include human methyltransferase (MTase)-like protein 3 (METTL3), MTase-like protein 14 (METTL14) and their additional subunits wilms tumor 1-associated protein (WTAP), vir like m6A methyltransferase-associated (VIRMA), zinc finger CCCH-type containing 13 (ZC3H13), cbl proto-oncogene like 1 (CBLL1) and RNA-binding motif protein 15/15B (RBM15/15B). Fat mass and obesity-associated protein (FTO) and alkB homolog 5 (ALKBH5) were identified as erasers on mRNA, and another eraser alkB homolog 3 (ALKBH3) was found to preferentially demethylate m6A in tRNA. M6A reader, including YT521-B homology (YTH) domain-containing proteins, heterogeneous nuclear ribonucleoprotein C/G (HNRNPC/G), heterogeneous nuclear ribonucleoprotein A2B1 (HNRNPA2B1), insulin-like growth factor 2 mRNA binding protein 1–3 (IGF2BP1-3) and FMRP translational regulator 1 (FMR1), utilize their functions to perform different mechanisms by binding with m6A-containing RNA [4, 5].

There are five members of the human YTH family: YTH N6-methyladenosine RNA-binding protein 1–3 (YTHDF1-3, also called DF1-3), YTH domain-containing 1 (YTHDC1/YT521-B, also called DC1), and YTH domain-containing 2 (YTHDC2, also called DC2). DF1-3 and DC2 mainly locate in the cytoplasm, while DC1 particularly locates in the nucleus. Although these five proteins share the highly conserved YTH domain, their function range appear to be distinct and interrelated. In fungi (Pho92, Mmi1), higher eukaryotes, plants, and other organisms, YTH structure is highly conserved. The conserved YTH structure of these homologous proteins indicates their crucial role in the biological processes of cell life. YTH proteins regulate almost all phases of m6A-modified RNA metabolism, including pre-mRNA splicing, RNA export, and translation. In addition, recent studies have found that YTH proteins regulate the function of noncoding RNAs, which means that YTH proteins regulate cell function by controlling the metabolism of noncoding RNAs. Due to their multiple functions in RNA metabolism, the dysfunction of these YTH proteins will play an essential role in many cancers.

In recent years, YTH family proteins have become important molecules in m6A associated epigenetic carcinoma. Various studies have shown that different YTH proteins play different functions in tumorigenesis. However, whether they are oncogenes or tumor suppressor genes remains controversial. Moreover, YTH proteins also play an important role in tumor metabolism, mitotic process, and immunity. In this review, we first introduce their structure and functions in m6A-modified RNA metabolism, including mRNA and noncoding RNA. We then focus on the role of YTH proteins in tumorigenesis and systematically elucidated their different functions in cancer.

## Structure of YTH domain-containing proteins

**Fig. 1:**
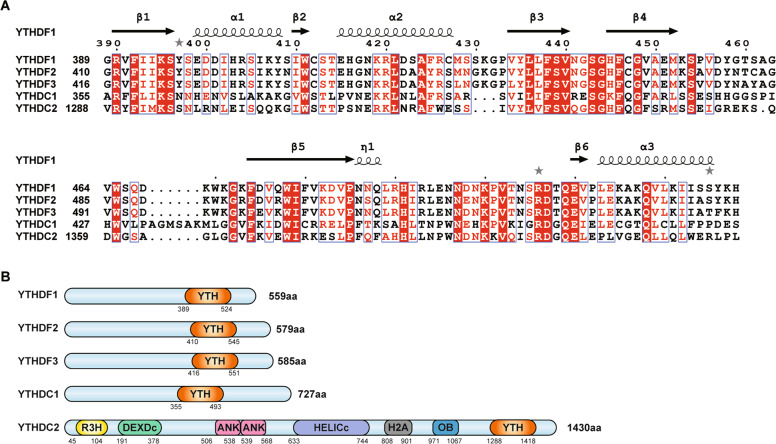
The structure of human YTH domain-containing proteins. **A** Multiple sequence alignment of YTH domains of human YTHDF1-3, YTHDC1-2 by ESPript3. **B** The domain composition of YTHDF1-3, YTHDC1-2. The five proteins YTHDF1, YTHDF2, YTHDF3, YTHDC1, and YTHDC2 share a highly conserved YTH domain (Orange). YTH domain is located at the C-terminal region.

Found in 174 different proteins in eukaryotes, the YTH domain, which is highly conserved and consists of 145 amino acids, is a sequence between the unstructured N-terminal and C-terminal [6] (Fig. 1B). Among DF1, DF2 and DF3 proteins, 86% of the corresponding YTH structural domain had the same sequence. The crystal structure of YTH family proteins showed us a new understanding of a common and different functions of these five human proteins (Fig. 1A). The secondary structure of the YTH domain consists of four α helices and six β chains, selectively binding to single-stranded, non-structured RNA [7]. YTH domain-containing proteins recognize the m6A-binding sites through a conserved aromatic cage consisting of three tryptophan residues.

The YTH domain of DF1 consists of β1, α1, β2 from the C termini, α2 from the N-terminus, and the loop between β4 and β5. DF1 specifically recognizes m6A sites using m6A-binding pocket composed of Trp411, Trp465, and Trp470 [8]. DF2 consists of three α helices, eight β strands, and two 3_10_ helices. The hydrophobic core consists of a β-barrel fold (β8–β1–β3–β4–β5–β2) and three α helices. Trp486 from the β4–β5 loop, Trp432 from the β2 strand and Trp491 from the β4–β5 loop are the basis of aromatic cage and can recognize m6A mononucleotide sites [9]. Residues W432 and W486 in the hydrophobic pocket contribute to the specific recognition of m6A. Researchers found that when W432 and W486 were mutated, the binding effect was significantly reduced. DF2 has been reported to preferentially bind to the conservation motif of G(m6A)C [9].

DC1 was showed a preference for C at the +1 position relative to m6A [10]. DC1 most frequently binds to the motif GG(m6A)C, while other DF1, DF2, DF3, and DC2 bind to G(m6A)C motif and A(m6A)C motif [8]. The DC1 aromatic pocket is composed of Trp411, Trp465, and Trp470, which can accommodate the m6A site. W377, W428 and L439 of the YTH domain of DC1 bind to methyl groups of m6A [11]. The YTH domain of DC1 also binds to single-stranded(ss) DNA containing m6A, and the binding affinity between DC1 and m6A ssDNA is higher than that of the corresponding sequence of ssRNA. In contrast, other proteins such as DF1 and DF2 showed weaker binding strength with ssDNA.

For DC2, Xu et al. found that α1, α2, and coils before α1, α2 and β4 could form m6A-binding pocket, which was consistent with the YTH domain of YTHDC1. Three amino acids W1310, W1360, and L1365 in DC2 are responsible for binding methyl groups of m6A [8, 12]. The coils between β2, β3 and α3, α4 form a positively charged surface around the m6A-binding pocket in the YTH domain of DC2. DC2 is unique because it contains several annotation domains and a common YTH domain, namely an R3H domain and a DEAH-box helicase core domain punctuated by two ankyrin repeats (ANK).

## General functions of YTH domain-containing proteins

### RNA transcription

Several studies revealed that DC1 and DC2 are involved in the efficiency and maintenance of RNA transcription. DC1 is located in the YT bodies, which contains the transcription focus. Chen et al. found that transcription derepression of many 2C-related retrotransposons was observed in mouse DC1 deficient embryonic stem cells (ESCs), partly caused by the loss of the LINE1-NCL-KAP1 complex [13]. Liu et al. found that DC1 is required to maintain the identity of mouse ESCs and retrotransposon repression by identifying a subset of transposable elements (TEs)-derived transcripts labeled m6A and then recruiting SET domain bifurcated histone lysine methyltransferase 1 (SETDB1) [14]. DC2 has an essential role in the successful meiotic of mammalian species as an m6A reader and 3′→5′ RNA helicase. Therefore, DC2 plays an essential role in maintaining transcription in the male germline [15].

### Pre-mRNA splicing

**Fig. 2:**
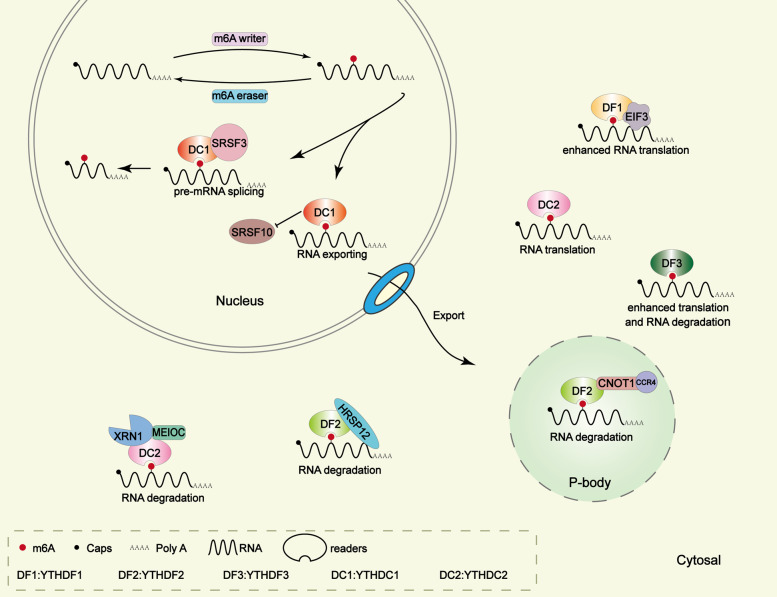
The detailed molecular mechanism of m6A reader protein YTH domain family on RNA. YTH domain-containing proteins participate in multiple steps of RNA metabolism including splicing, export, translating, degradation and so on.

Almost 95% of human genes undergo alternative splicing, which enables them to perform different regulatory functions. DC1 is the only YTH domain-containing protein localizes to nuclear speckles in cultured mammalian somatic cells. DC1 directly regulates the splicing of mRNA by connecting trans- and cis-regulatory elements and changes alternative splicing patterns in a concentration-dependent manner [16]. Xiao et al. found that DC1 regulated the splicing of mRNA by recruiting and regulating pre-mRNA splicing factors (SR-rich splicing factor 3, SRSF3, and SRSF10) into the binding region of targeted mRNA, so as to play the function of splicing factors (Fig. 2). The study found that both SRF3 and SRF10 compete for the binding of the N-terminal domain of DC1, which determines different alternative splicing results. DC1 promotes the exon intron of targeted mRNA by facilitating SRSF3 and blocks SRSF10 mRNA binding [17]. The interaction between DC1 and SRSF10 can promote target mRNA exon skipping [18]. DC1 was associated with processing factors (CPSF6, SRSF3, and SRSF7) at the 3′ end of pre-mRNA and regulated alternative polyadenylation. DC1 deficiency leaded to massive alternative splicing defects in oocytes, which can be rescued by supplying DC1 [19]. Luxton et al. found that the interaction between DC1 and metadherin promoted the alternative splicing of tumor-associated proteins, such as BRCA, MDM2, and VEGF [20]. Gao et al. found that the presence of DC1 led to splicing of m6A-modified Titin mRNA, suggesting the cardiac biological function and the development of dilated cardiomyopathy (DCM) [21]. In addition to pre-mRNA splicing, m6A-methylated circRNA and lncRNA are also regulated by DC1 reader. In HBV-positive hepatocellular carcinoma (HBV^+^ HCC), DC1 bound to m6A-modified circ-ARL3 and favored its reverse splicing and biogenesis. The presence of circ-ARL3 in HBV^+^ HCC can promote the growth of tumor cells [22]. The formation of HSATIII lncRNA was induced by stress. m6A-modified HSATIII was found to sequester DC1 in nuclear stress bodies (NSBs) during thermal stress recovery. The results showed that DC1 regulates splicing the m6A-modified HSATIII [23].

### RNA export

Although DC1 exists in both cytoplasm and nucleus, studies have shown that DC1 mainly plays a role in the nucleus [24–27]. DC1 mediates the transport of m6A-methylated mRNAs from nuclear to the cytoplasm. Overexpression of DC1 leads to the decrease of m6A mRNA in the nuclear, while inhibition of DC1 resulted in the accumulation of m6A mRNA in the nuclear and the decrease of the m6A level in the cytoplasm [26]. m6A-modified mRNA can be exported in a variety of ways. m6A mRNA was recognized by DC1 and delivered to the nuclear RNA export factor (NXF1) via association with the SRSF3, a member of the SR protein family [28] (Fig. 2). NXF1 is an export receptor that binds SRSF3. DC1 can facilitate the binding of NXF1 and SRSF3, thus promoting the exportation of methylated mRNA [26]. Another mRNA export function was mediated by the transcription and export complex (TREX). TREX played an important role in the recruitment of DC1 to mRNA [29].

### mRNA translation

In eukaryotic cells, the control of mRNA translation is essential for gene regulation. There are two ways involved in the process of mRNA translation: cap-dependent and -independent translation. The cap-dependent translation begins with the recognition of m7G-cap, followed by the recruitment of the 40S ribosomal subunit to the 5′ terminus. The cap-independent approach requires an internal ribosomal entry site (IRES). Although most cellular mRNA use cap-dependent mechanisms, the initiation of a few mRNAs is mediated by IRESs [30]. In YTH family, DF1, DF2, DF3, and DC2 proteins are identified as translation regulatory proteins and regulate a variety of mRNA targets proteins. DF1 enhances the translation efficiency of target transcripts in two ways: first, it accelerates the occupancy of the ribosome to methylated mRNA; second, it interacts with initiation factors to promote expression of target mRNA. DF2 was found to regulate the translation of m6A-modified mRNA by cooperating with DF1 or by individually binding to its m6A sites on its 3′UTR [31, 32]. DF3 has been reported to primarily regulated the initiation of m6A-methylated mRNA translation by interacting with DF1 in cytoplasmic [33]. DC2, which contains the RNA helices domain, promoted structured mRNAs translation by resolving its secondary structures [34].

DF1 was found to be a major translation regulator in YTH proteins. Its effect on translation has been widely studied. A proposed DF1 related mRNA translation mechanism model is the eIF4G-mediated loop structure and the interaction between DF1 and eIF3. For instance, DF1 directly binds to the m6A-modified EIF3C to improve its translation efficiency, thereby facilitating the occurrence and metastasis of ovarian cancer [35]. DF1 functions its role in different m6A-modified mRNA sites. Li et al. found that DF1 binding to eEF2 regulates the m6A-induced pyruvate dehydrogenase kinase 4 (PDK4) mRNA translation elongation by interacting with 5′UTR, but has no effect in 3′UTR. Moreover, the effect of DF1 overexpression increase PDK4 translation only exist in the 5’UTR region, while attenuated for 5′UTR mutated [36]. However, it has also been reported that DF1 binds to m6A sites in the CDS region of Snail mRNA rather than 3′UTR, and enhances trigger polysome-mediated translation elongation by recruiting the translation elongation factor eEF2 [37]. DF1 acts as a double-edged sword in tumor phenotype. It can not only promote the translation and expression of tumor promoter genes, but also the translation and expression of tumor suppressor genes. On the one hand, DF1 promotes the occurrence and metastasis of tumors. For example, YTHDF1 promoted the translation of β-catenin mediator TCF7L2/TCF4 (Wnt signaling components) in the Wnt/β-catenin signaling pathway. Therefore, DF1 was indispensable in physiological cell development and Wnt-driven tumorigenesis [38]. On the other hand, DF1 plays a role as a tumor suppressor in many other cancers. DF1 promoted the translation of methylated HINT2 mRNA, one of the ocular melanoma tumor suppressor genes [39]. Researchers have found that only the truncated DF1 (aa100–200) retained its translational activation, suggesting that aa100–200 is responsible for binding to the translation mechanisms [40].

Several studies have found that DF2 can regulate the translation of m6A-modified genes. Shmakova et al. found that PBRM1 could cooperate with DF2 to improve the translation level of m6A-modified HIF-1α mRNA [41]. Sheng et al. found that DF2 individually acts as a tumor promoter to enhance tumor growth via facilitating 6PGD mRNA translation [31]. DF2 also plays its translation regulation function through synthetizing with DF1, and regulates the Wnt5a pathway by promoting the local translation of GC axons, so as to control the development of cerebellar parallel fibers [32]. Shi et al. demonstrated for the first time that DF3 promoted mRNA translation by interacting with ribosomal 40S/60S subunits. DF3 binds tightly to RNA targets first, then promotes the binding of DF1, and further promotes the activity. In addition, DF3 significantly improves the translation efficiency of DF1/3 common targets mRNA, but does not improve the unique targets of DF3, indicating that DF3 serves as a fine tuner and facilitates the translation of DF1 on m6A-modified RNA [33]. Among YTH domain proteins, DC2 is the only protein containing RNA helices domain, which promotes the structured translation of mRNA by resolving secondary structures. Due to the high TE of CDS, m6A-modified CDS regions is likely to form a more stable secondary structure. DC2 releases the translation elongation efficiency of m6A-modified CDS domain which was reduced by secondary structure [34]. DC2 can enhance translation efficiency of methylated mRNA in mammalian cells, and destroy transcription after translation, enabling normal prophase I of meiotic [42]. The underlying mechanisms of m6A-modified mRNA translation needs to be further studied.

### RNA stability and degradation

A number of studies have found that there is a correlation between YTH proteins and mRNA stability. In oral squamous-cell carcinoma (OSCC), DF1-mediated c-Myc stability in an m6A-dependent manner [43]. The methylation of MAT2A mRNA by METTL16 was stabilized by DC1 [44]. In addition to YTHDF1-3, YTHDC2 also interacts with multiple meiotic prophase-related RNA, such as RNA transcript cyclin A2 (CCNA2), to control spermatocyte state by regulating of RNA stability [45]. For lncRNA, DF1 and DF2 promoted the stability of lncRNA THOR in the m6A-dependent regulation, which provide a new way to explore the metabolic mode of m6A-modified noncoding RNA [46].

As for the m6A-modified mRNA degradation, DF2 mainly promoted the decay of m6A-methylated mRNA, and DF3 served as an assistant. Evidence have shown that the m6A/A ratio of mRNA from translating pool was significantly increased after DF2 downregulation, indicating that DF2 plays the most important role in the degradation of m6A-modified mRNA [47]. DF2 can transport m6A-modified mRNA from the translation pool to the P bodies. P bodies are a kind of non-membranous compartment in the cytosol and a kind of conserved RNP granules, which play an essential role in mRNA storage and standard cellular process [48]. Proteins involved in mRNA degradation are enriched in the P bodies. The decay of m6A-containing RNA is initiated by deadenylation, which is the first step of mammalian mRNA decay and promotes the formation of P bodies [48].

Recent studies have shown two effective DF2-dependent m6A-modified mRNA degradation mechanisms: the YTHDF2-CCR4/NOT axis and the YTHDF2-HRSP12-RNase P/MRP axis. The CCR4/NOT complex is the second major eukaryotic deadenylase, which contains nine subunits. CAF and CCR4A are two deadenylases subunits of the CCR4/NOT complex, and CNOT1 is a large scaffold subunit [49]. The C-terminus of DF2 recognizes m6A, and the N-terminus promotes deadenylation by directly binding to the superfamily homology (SH) domain of CNOT1. DF2 directly recruited the CCR4/NOT deadenylase complex into m6A-containing mRNA, independent of the association between DF2 and P body components [50]. About the YTHDF2-HRSP12-RNase P/MRP axis, RNase P and RNase MRP are closely related and play an essential role in endoribonucleases. HRSP12 selectively binds to the minimal region of 1–100 amino acids at the N-terminal of DF2 and is sufficient for rapid RNA degradation with the help of DF2. DF1 and DF3 can also be combined with HRSP12, but weaker than DF2 [51]. HRSP12 mainly binds to approximately 862 nucleotides upstream of DF2-binding sites of m6A mRNAs, among which GGUUC is the most predominant RNA motif of HRSP12, and RNase P/MRP mostly cleaves approximately 421 nucleotides downstream of a DF2-binding site within m6A RNAs [51]. Together, the C-YTHDF2 and the N-YTHDF2 both lead to the function of decay relative mRNA abundance. In glioblastoma (GBM), DF2 promotes the m6A-dependent mRNA decay of LARA and HIVEPS, which was related with the patient survival. Furthermore, DF2 also promotes the occurrence of GBM via downregulating the target mRNA downstream of EGFR [52]. The underlying mechanism of autophagy is related with Atg5 and Atg7 mRNA. Wang et al. found that DF2 can capture the m6A-methylated Atg5 and Atg7, leading to mRNA degradation and reduced protein level, thereby alleviating the possibility of autophagy [53]. More other studies have firmly demonstrated that DF2 can regulate the m6A-modified RNA through RNA-degradation mechanism [54–58]. In colorectal cancer (CRC), lncRNA XIST was downregulated by the m6A-DF2-dependent pathway [59].

DF3 shares over 65% protein sequence identity with both DF1 and DF2. At present, it has been found that DF3 synergy with DF1 to promote protein synthesis, thus affecting m6A mRNA decay mediated by DF2 [33]. Recently, a new unified model has shown that DF proteins bind to the same m6A-modified mRNAs rather than different mRNAs, to perform multiple functions, such as mRNA degradation and cell differentiation. Only when the paralogs of three DF proteins are present at the same time can DF proteins played their collective action in regulating RNA stability and differentiation [60]. A study uncovered that DF3 can be regulated by the lncRNA GAS5-YAP axis and reduce the life span of GAS5 through m6A-mediated degradation in CRC [61].

m6A-methylated mRNA can also be destabilized through the DC2-mediated pathway. DC2 is the only YTH protein with a helicase domain and belongs to the DEAD/H-box family of ATP-dependent RNA helicases [62]. It is an RNA-induced ATPase with a 3ʹ/5ʹ RNA helicase activity. DC2 can regulate RNA levels during mitotic by binding with meiosis-specific protein (MEIOC). MEIOC and DC2 regulate mitotic cell cycle programming by interacting with mitotic cell cycle-associated transcripts. The 3′–5′ RNA helicase DC2 binds to MEIOC and simultaneously interacts with the 5′–3′ exoribonuclease XRN1. DC2 mediated the decay of specific m6A mRNAs through the helicase domain Ankyrin repeat, resulting in the destabilization of specific mRNAs [63]. DC2 can bind to the m6A-modified lipogenic genes mRNA, including Srebp-1c, FASN, Scd1, and Acc1, to decrease their mRNA stability and inhibit gene expression [64].

## Regulation of YTH domain-containing proteins expression in cancers

The expression of YTH domain-containing proteins in cancers is regulated by different mechanisms. It was demonstrated that smoking and hypoxia condition correlated with the expression level of YTH proteins. Hypoxia-mediated DF2 overexpression promotes cell proliferation and invasion by activating the mTOR/AKT axis [65]. Hypoxia is also the main regulator of DC1 mRNA expression pattern, which induces the transformation of DC1 mRNA into two non-protein-coding mRNA variants [66–68]. Yu et al. showed that histone H3 lactylation at lysine 18 (H3K18la) activated the transcription of DF2 in ocular melanoma [69]. Nishizawa et al. found that c-Myc induced the transcriptional activity of DF1 in CRC [70]. Ni et al. showed that YAP upregulated the transcription of DF3. In addition, HIF-1α and TNF-α promote DC2 transcriptional activity by binding to DC2 promoter [62, 71]. It has also been reported that Musashi-1 (MSI1) positively regulates the expression of DF1 by stabilizing DF2 mRNA in GBM cell [72]. Some microRNAs, including miR-3436, miR-376c, miR-139-3p, etc., have been proposed to suppress DF1, DF2, and DF3 by targeting the mRNA of DF1, DF2, and DF3 in various cancers [68, 73–84].

In addition, YTH family proteins are also regulated at the protein level. Fang et al. showed that ERK1/2 regulates the stability of DF2 protein by inducing the phosphorylation of serine 39 and threonine 381 of DF2, which further promoting the invasion growth of glioblastoma [52]. In contrast, in ovarian cancer, FBW7 directly binds to and negatively regulates the protein level of DF2 via ubiquitination-mediated proteolytic degradation [85].

## YTH domain-containing proteins function as m6A readers in cancers

In recent years, increasing evidence shows that YTH family proteins play a key role in cancer progression as m6A reader. YTH domain-containing proteins are closely related to the hallmarks of cancer, including cell cycle, apoptosis, metastasis, cell metabolism, and immunity. We will first discuss the role, expression, and clinical characteristics of YTH domain-containing proteins in a variety of cancers, and then describe their functions in cancer progression.

### The role, expression, and clinical characteristics of YTH domain-containing proteins in cancers

**Fig. 3:**
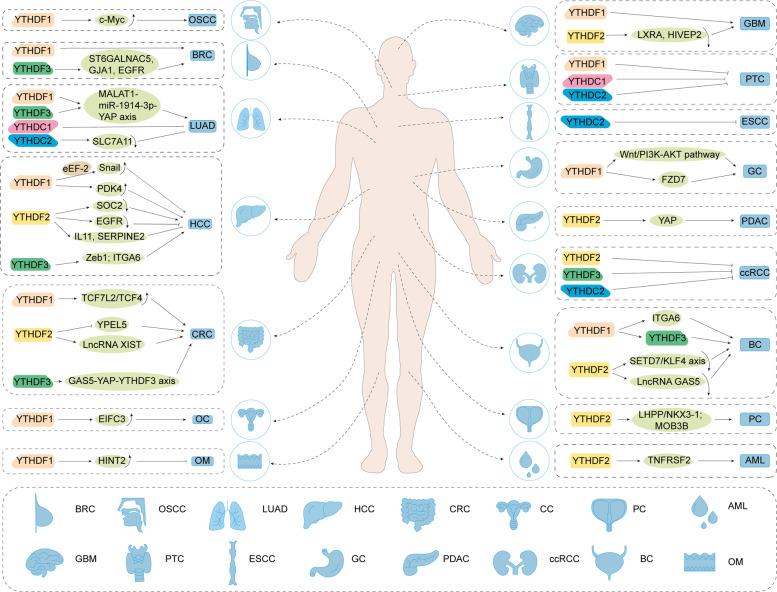
YTH family proteins in various tumorigenesis. The momentous mechanical pathways of YTH domain proteins involved in human cancers.

Many studies have discovered that m6A modification in RNA is an essential regulator in various cancers. As the most essential m6A readers, YTH family proteins are key molecules in various tumorigenesis (Fig. 3). They regulate levels of oncogenes or tumor suppressor genes by “reading” m6A-modified RNA, and then the target genes perform different functions in cancer. The summary of YTH family proteins’ direct mechanisms in various types of cancers is listed in Table 1.

**Table 1:** Oncogenic/tumor-suppressive role of YTH domain-containing proteins in various human cancers.

Proteins	Role in diseases	Cancer	Intersection molecules and/or pathway	References
YTHDF1	OG/TS	LUAD	YAP; MALAT1-miR-1914-3p-YAP axis	[104, 113]
YTHDF1	OG	GC	Wnt/PI3K-AKT pathway; FZD7-β-catenin axis	[110, 111]
YTHDF1	OG	BC	ITGA6	[105]
YTHDF1	OG	CRC	TCF7L2/TCF4, c-MYC; Wnt/β-catemin	[38, 70, 144]
YTHDF1	OG	GBM	hsa-mir-346	[78]
YTHDF1	OG	CC	PDK4	[36]
YTHDF1	OG	HCC	FZD5	[111]
YTHDF1	TS	OM	HINT2	[39]
YTHDF1	OG	OSCC	METTL3/m6A/ YTHDF1/c-Myc axis	[43]
YTHDF1	OG	OC	EIFC3	[35]
YTHDF1	OG	PC	TRIM44	[145]
YTHDF2	OG/TS	LUAD	YAP	[113]
YTHDF2	OG	BC	SETD7/KLF4 axis	[57]
YTHDF2	OG	CC	LncRNA GAS5	[146]
YTHDF2	OG	CRC	LncRNA XIST, YPEL5; SOX4	[59, 147]
YTHDF2	OG	GBM	EGFR/SRC/ERK pathway	[52]
YTHDF2	TS	HCC	SOC2, EGFR, IL11, SERPINE2	[101, 112]
YTHDF2	OG	PDAC	YAP	[148]
YTHDF2	OG	AML	TNFRSF2	[149]
YTHDF2	OG	PC	METTL3/YTHDF2/LHPP/NKX3-1; MOB3B	[56, 74, 75]
YTHDF3	OG	BC	ITGA6	[105]
YTHDF3	OG	BRC	ST6GALNAC5, GJA1, EGFR	[130, 150]
YTHDF3	OG	CRC	GAS5-YAP-YTHDF3 axis;	[146]
YTHDF3	OG	HCC	Zeb1; ITGA6	[132, 151]
YTHDF3	OG	LUAD	MALAT1-miR-1914-3p-YAP axis	[104]
YTHDC1	OG	UCEC		[152]
YTHDC2	TS	LUAD	SLC7A11	[153]
YTHDC2	TS	CRC	HIF-1α	[154]

*OG* oncogene, *TS* tumor suppressor, *LUAD* lung adenocarcinoma, *GC* gastric cancer, *BC* bladder cancer, *CRC* colorectal cancer, *GBM* glioblastoma, *CC* cervical cancer, *HCC* hepatocellular carcinoma, *OM* ocular melanoma, *OSCC* oral squamous-cell carcinoma, *OC* ovarian cancer, *PC* prostate cancer, *PDAC* pancreatic ductal adenocarcinoma, *AML* acute myeloid leukemia, *BRC* breast cancer, *UCEC* uterine corpus endometrial carcinoma.

In recent years, a number of cohort studies from TCGA patient samples and other online databases illustrated the expression level of YTH family and its association with the clinical characteristics of some cancer types [86–97]. We summarized the association and clinical characteristics between YTH family members and various tumors in Table 2.

**Table 2:** Expression of YTH domain-containing proteins in various human cancers.

YTH family proteins	Cancer type	mRNA/protein level change	Expression level in patients (high/low)	Clinical characteristics	References
YTHDF1	LUAD	mRNA protein	High High	Associate with better OS and RFS; p53 mutation	[99, 155]
YTHDF1	CRC	Protein	High	Associate with KRAS and BRAF mutation, gender in COAD; associates with KRAS mutation	[93]
YTHDF1	HNSCC	mRNA	High	–	[92, 156]
YTHDF1	HCC	mRNA protein	High High	Positive with pathology stage; correlate with OS, shorter DFS rate; independent poor prognostic factor	[90, 157–159]
YTHDF1	ESCA	mRNA	High	–	[95]
YTHDF1	PAAD	protein	High	Mutations	[94]
YTHDF1	PTC	mRNA	Low	–	[68]
YTHDF1	BRC	mRNA protein	High High	Poor survival	[92]
YTHDF1	UCEC	mRNA	High	Correlate with prognosis	[160]
YTHDF1	ESCC	protein	High	Correlate with OS	[87]
YTHDF2	LUAD	mRNA	High	Better OS rate and RFS rate; p53 mutation	[99, 155]
YTHDF2	CRC	Protein	High	BRAF mutation in READ	[93]
YTHDF2	HNSCC	mRNA	High	–	[92, 156]
YTHDF2	HCC	mRNA	High	Correlate with OS and prognosis	[157]
YTHDF2	ESCA	mRNA	High	Correlate with better survival	[95]
YTHDF2	PAAD	mRNA	High	–	[94]
YTHDF2	BRC	mRNA protein	High High	–	[92]
YTHDF2	UCEC	mRNA	High	–	[160]
YTHDF3	LUAD	protein	High	Worse OS.	[91, 98]
YTHDF3	CRC	mRNA protein	Low High	BRAF mutation in READ	[93]
YTHDF3	HCC	–	–	Correlate with shorter DFS rate	[90, 157, 158]
YTHDF3	PAAD	protein	High	–	[94]
YTHDF3	BRC	protein	High	Poor survival and RFS rates	[92]
YTHDF3	HNSCC	mRNA	High	–	[92]
YTHDF3	ESCC	–	–	Correlate with OS	[87]
YTHDC1	LUAD	mRNA	Low	–	[99]
YTHDC1	CRC	mRNA	High	Correlate with TNM stage in COAD; BRAF mutation in READ	[93]
YTHDC1	ESCA	mRNA	High	–	[95]
YTHDC1	PTC	mRNA	Low	–	[68]
YTHDC1	BRC	mRNA	Low	–	[92]
YTHDC1	HNSCC	mRNA	High	Correlate with better OS	[92]
YTHDC1	UCEC	mRNA	Low	–	[160]
YTHDC1	OS	–	–	Correlate with OS	[96]
YTHDC2	HCC			Negatively correlated with the prognosis	
YTHDC2	LUAD	mRNA	Low	Correlate with tumor stage	[99]
YTHDC2	HNSCC	mRNA	Low	Correlated with better OS and clinical outcome	[92, 156]
YTHDC2	ESCA	–	–	Gender and TNM stage	[95]
YTHDC2	PTC	mRNA	Low	–	[68]

*LUAD* lung adenocarcinoma, *CRC* colorectal cancer, *HNSCC* head and neck squamous-cell carcinoma, *HCC* hepatocellular carcinoma, *ESCA* esophageal cancer, *PAAD* pancreatic adenocarcinoma, *PTC* papillary thyroid carcinoma, *BRC* breast cancer, *UCEC* uterine corpus endometrial carcinoma, *ESCC* esophageal squamous-cell carcinoma, *OS* osteosarcoma.

TCGA database, GEO database, real-time qPCR results, and IHC scores from clinical patient samples showed the expression level of the YTH family was correlated with different cancers. Studies found that YTH family members are highly expressed in a variety of tumors, and are associated with cancer prognosis, such as lung adenocarcinoma (LUAD), CRC, hepatocellular carcinoma (HCC), pancreatic adenocarcinoma (PAAD), esophageal squamous-cell carcinoma (ESCC), head and neck squamous-cell carcinoma (HNSCC) and so on. Conversely, DF1 decreased in PTC and DC1 decreases in LUAD. Interestingly, DF3 expression level is reduced in mRNA level while upregulated in protein aspects in CRC. DF1 mRNA or protein expression level in LUAD, HCC, and ESCC were positively correlated with better prognosis rates, such as the overall survival rates (OS), relapse-free survival (RFS), and disease-free survival (DFS) [98, 99]. The combination of YTHDF1 and METTL3 can be used as a biomarker to evaluate the prognosis and reflect the degree of HCC malignancy [100]. The overexpression of DF2 in HCC contributes to better OS and prognosis. While overexpressed DF3 protein resulted in worse OS rates of LUAD, DF3 was correlated with shorter DFS rates in HCC, OS in ESCC and poor survival and RFS rates in BRC. The higher mRNA level of DC1 and DC2 were correlated with better OS rates in HNSCC, and DC1 was also associates with OS in osteosarcoma. From the aspect of pathology, DC1 overexpression in CRC, DC2 overexpression in LUAD and PTC were positively correlated with TNM staging, suggesting that YTH members have the potential to distinguish the degrees of pathological classification of tumors.

### The interaction between YTH domain-containing proteins and m6A writers or erasers

m6A modification is a dynamic and reversible process regulated by m6A writers, erasers and readers. The dysregulated m6A modification and its related regulatory proteins were found to play significant roles in cancers. Therefore, it is essential to understand the interaction between YTH domain-containing proteins and m6A writers and erasers.

Plenty of studies revealed that the cross-talk among m6A-related proteins could regulate cancer growth and progression. Chen et al. found that METTL3 regulates the expression level of tumor suppressor gene SOCS2 through recruiting DF2 to the 3′end of SOCS2 transcript, and mediated the decay of the SOCS2 mRNA, resulting in tumor promotion effect in liver cancer [101]. METTL14 influences tumorigenesis via regulating target mRNA degradation which was relied on the YTHDF2-dependent pathway [102]. Moreover, METTL3 and METTL14 regulate the degradation of target mRNA via recruiting DC2 or DC1 to the target mRNA [103]. Besides, METTL3 and METTL14 directly enhance the translation of target RNA by recruiting DF1 or DF1/DF3 complex into translation initiation complex, which further indicates the adverse or reverse progression of tumor [104, 105]. In contrast, m6A erasers ALKBH5 and FTO decrease m6A modification of the target genes, thus inhibiting the direct binding of DF2 and DF1 in the target RNA, which further influence the translation or degradation efficiency of corresponding tumors [106, 107]. In addition, Subbarayalu et al. unveiled a novel function for m6A writer–eraser–reader axis in regulating tumorigenesis. METTL14/ALKBH5 can determine the m6A status of target genes by regulating the expression of each other and inhibiting the expression level of DF3, so as to play their tumorigenic role [108]. In brief, m6A writers and erasers facilitate carcinogenesis by recruiting m6A readers, and play their respective roles in different tumors.

### Cell cycle-tumor proliferation and tumorigenesis

Cell cycle is the most essential pathway to regulate tumorigenesis. Cell cycle checkpoint regulators have been considered to be important regulators of G0/G1 cell cycle transition. Cyclins, CDKs and CKIs are components of the cell cycle pathway. DF1, as an oncogene, has been reported to increase G0/G1 cells by regulating CDK2, CDK4, and cyclin D1 translational efficiency in non-small cell lung cancer (NSCLC). DF1 regulates cell cycle mainly through regulating relative mRNA translation in different cancers. When DF1 is deficient, NSCLC cell proliferation and xenograft tumor formation is inhibited [109]. In addition, DF1 also promotes the proliferation and tumorigenesis of gastric cancer cell in an m6A-dependent manner by enhancing the translation efficiency of key Wnt receptors frizzled7 (FZD7) and frizzled5 (FZD5). DF1 can activate the Wnt/β-catenin pathway by controlling the translation of the key receptor FZD7 [110, 111]. DF1 also controls the stability of c-Myc in oral squamous-cell carcinoma (OSCC) [43].

Compared with DF1, DF2 mainly controls the progression of tumor cells by promoting the degradation of m6A-modified mRNA related to cell proliferation. In prostate cancer (PCa), the reduced DF2 protein level leads to the inhibition of PCa progression. Study indicates that DF2 mediates the degradation of LHPP and NKX3-1 mRNA level, which further diminishes cell proliferation via reducing AKT pathway [56]. In GBM, DF2 affects the mRNA expression of LXRA and HIVEP2 by altering the degradation of LXRA and HIVEP2 mRNA, and further affects GBM proliferation, tumorigenesis, and invasion [52]. Zhong et al. found that DF2 directly binds to the m6A-modified 3′UTR region of EGFR and promotes the decay of EGFR in HCC cells [112]. On the contrary, Fang et al. found that EGFR can maintain DF2 expression through EGFR/SRC/ERK pathway [52]. That gives us a new understanding of the feedback regulation of DF2 in EGFR dependent regulation. In NSCLC, DF1 and DF2 competitively bind DF3 in a m6A-independent manner and YAP pre-mRNA in a m6A-dependent modification manner. YTH proteins jointly regulate the expression level of YAP by playing m6A-related function. DF2 exerts its degradation ability by binding to YAP pre-mRNA. DF3 recognizes the m6A sites of YAP mRNA, and then DF1 binds to DF3 to promote the translation level of YAP mRNA. Thus, in NSCLC, YTH proteins play a positive tumor proliferation regulator in m6A-modified YAP [113]. Sheng et al. found that DC1 has a strong cancer-promoting effect via regulating the DNA replication complex in AML. DC1 enhances the self-renewal of leukemia stem cells (LSCs) through regulating MCM4, which is the critical regulator of DNA replication [114]. In LUAD, the overexpression of DC2 regulates CYLD/NF-κB signaling pathway and inhibits the proliferation of lung cancer cells [115].

Besides cell cycle checkpoint regulator, correct and regular mitosis is also very important for mammalian growth. Fei et al. found that DF2 promoted mitosis entry via negatively regulation of Wee1-like kinase. DF2 recognizes m6A modification and mediates the decay of WEE1 transcripts, leading to the timely entry of mitotic [116]. In addition, genomic instability in mammalian cells is regulated by DF2. R-loops are nucleic acid structures that correlate with the genome stability. The depletion of DF2 resulted in the increase of R-loops and the accumulation of m6A on RNA:DNA hybrids and cell growth retardation [117]. In mouse germ cells, DC2 expression is upregulated, facilitating the transition from mitosis to meiosis. The expression levels of mitotic transcripts, such as CCND2 and others, were upregulated by DC2 in an m6A-dependent manner [45].

In conclusion, DF1, DF2, DF3 broadly play the role of tumor promoter in a variety of tumors, and DF2 and DC2 play a role in promoting cell mitosis. While there are little studies of DC1 over cancer cells proliferation function so far, thus more specific functions and mechanisms need to be further explored.

### Apoptosis-resisting tumor cell death

Apoptosis plays an important role in animal development. There are two kinds of apoptosis pathways in the mammalian system, namely the intrinsic pathway (also called the mitochondrial pathway) and the extrinsic pathway [118]. The intrinsic apoptotic pathway is activated by various intracellular stimuli, and relies on the formation of apoptosome, which are composed of procaspase-9, apoptotic protease-activating factor 1 (Apaf-1), cytochrome c, and a series of Bcl-2 family members, such as Bax, Bak, Bcl-2, and Bcl-x. Apoptosis is also activated by some related apoptosis modulators, such as the JNK, TGF-β, and MMP pathways [119].

From the perspective of apoptosis, the occurrence of tumor is due to the obstruction of apoptosis. BINP3 is a pro-apoptosis gene associated with apoptosis genes Bcl-2 and caspase 3. Studies have found that DF2 was identified as the promoter of breast tumor through inducing the degradation of the tumor suppressor gene BINP3 mRNA on its 3′UTR in the progression of breast tumor [120]. In addition, another study found that in MYC-driven breast cancer, DF2 induced apoptosis in human triple-negative breast cancer (TNBC) cell lines and hindered the growth of xenografted tumor in vivo [121]. In LUAD cells, circASK1 is closely correlated with the activation of ASK1/JNK/p38 signaling pathway, leading to the activation of ASK1-induced apoptosis. In addition, DF2 mediates the cleavage of m6A-modified circASK1 in gefitinib-resistant cells, thereby reducing the enrichment of circASK1 and reducing apoptosis of tumor cell [122]. In HNSCC tumor tissues, GSEA analysis suggested that the high expression of DC2 was associated with apoptosis-related genes capspase3, 6, 8, 9, Bid, UNE2S, and HERC3 [123].

Besides affecting apoptosis-related genes and pathways, there are many possible methods to detect and visualize undergoing apoptosis in cells and tissue. For example, TUNEL assay reveals that DF2 knockout (KO) cells lead to increased apoptosis rates, and flow cytometry analysis showed that the reduction of DF2 affected G2/M transition [124]. Wang et al. found that DF1-KO aggravates the apoptosis effects via regulating RANBP2 mRNA in an m6A-dependent manner in CRC cells [125]. In epithelial ovarian cancer (EOC), DF2 regulates cancer cells apoptosis (detected by flow cytometry analysis) via the double-negative feedback loop with miR-145 [76]. In short, YTH family members, especially the DF2 protein, play the role in reducing apoptosis in cancer cells.

### Metastasis-tumor invasion and metastasis

Cancer metastasis is the process of cancer cells spreading from the primary sites to the distal organs, and it is the main cause of cancer death. Epithelial–mesenchymal transition (EMT) is an important step in cancer cell metastasis. Snail mRNA is one of the key transcription factors of EMT. Lin et al. found that DF1 functions as the translation promoter of m6A-modified Snail mRNA in CDS in cancer cell, and therefore drives metastasis [37]. In gastric cancer, DF1 facilitates tumor cell migration via promoting USP14 mRNA translation [126]. DF1 is involved in m6A-induced KRT7 mRNA translation in lung metastasis of breast cancer [127].

The Wnt/β-catenin pathway is closely related to metastasis. AXIN1 is a negative regulator of the Wnt/β-catenin pathway. DF2 is upregulated in LUAD and functions as a migration promoter via downregulating AXIN1 expression. Thus, DF2-catenin AXIN1-Wnt/β-catenin is a new pathway that favors the migration in lung cancer cells [128]. In CRC, lncRNA XIST has been identified as an oncogene and induces cell migration. DF2 reversed the metastasis progression via mediated degradation of XIST [59]. Similarly, DF2 also facilitates tumor metastasis of CRC through accelerating the decay of YPEL5 mRNA in an m6A-dependent manner [129]. Chang et al. reported that DF3 promotes breast cancer brain migration via stimulating the translation of metastasis-related genes, such as STAGALNAC5, GJA1, EGFR, and VEGFA. Among the key brain metastatic gene transcriptions, ST6GALNAC5, GJA1, EGFR, VEGFA, and SRC transcripts are enriched in the m6A peak, and have already been identified as the key transcripts associated with metastasis [130]. DF3 also regulates cell migration and invasion via promoting m6A-modified YAP mRNA translation. DF3, DF1, and eIF3b were recruited into the translation initiation complex to promote the stability of YAP mRNA in NSCLC cells [104].

LncRNA metastasis-associated lung adenocarcinoma transcript 1 (MALAT1), which localized in nuclear speckles (NSs), was significantly associated with metastasis progression. In cancer cells, DC1 promotes cell migration through recognizing and binding m6A-modified MALAT1 in NSs, resulting in the accumulation of metastasis-related genes [131]. Zeb1 is also a key factor in cell migration. In HCC, DF3 enhances Zeb1 mRNA stability in an m6A-dependent manner [132]. In lung cancer, DC2 functions as a tumor suppressor. Reduced DC2 expression leads to increased lung cancer migration via the CYLD/NF-κB signaling pathway [67].

### Cellular metabolism in cancers

Metabolism abnormalities are a major hallmark of cancer. Aberrant cancer metabolism is related with increased aerobic glycolysis, lipid metabolism as well as hypoxia. Hypoxia occurs mainly in solid tumors. The hypoxia-inducible factor (HIF) family of transcription factors are mainly responsible for the cellular response to hypoxia. They consist of two components, HIF-1α and HIF-1β. Shmakova et al. found that DF2 cooperates with PBRM1 to promote m6A-modified HIF-1α mRNA translation [41]. In endometrial cancer, HIF-1α induces DC1 expression pattern under hypoxia. HIF-1α transforms DC1 isoforms 1 and 2 into two non-protein mRNA variants. This change is correlated with alternative splicing of downstream cancer-related genes [66]. Reactive oxygen species (ROS) is a by-product of mitochondria metabolism and hypoxia. Shi et al. found that DF1 is an evolutionarily activity-selected plateau adaption gene, which is amplified in various cancers, including NSCLC. Downregulation of DF1 in lung cells leads to ROS accumulation and reduces hypoxia adaption through the downstream Keap-Nrf-AKR1C1 axis [109]. Most cancers obtain their energy from the Warburg effect. Pyruvate dehydrogenase kinase 4 (PDK4) is involved in m6A regulated glycolysis and ATP production of cancer cells. The DF1/eEF2 complex directly recognizes and binds to the 5’UTR of m6A-modified PDK4, positively regulating its translation elongation and mRNA stability [36].

In leukemia, DF2-mediated translation promotion of two key glycolytic genes PFKP and LDHB can be reduced by R-2-hydroxyglutarate (R-2HG) [133]. In cervical cancer (CC), DF1 assists with METTL3 to enhance the stability of HK2 via m6A modification, thus promoting the Warburg effect of CC [134]. DF1 promotes MYC translation in an m6A-dependent method. Upregulated MYC leads to the subsequent glycolysis improvement and tumor cell proliferation [135]. Contrary to the previous promotion of the Warburg effect, Hou et al. showed that DC1 attenuated the Warburg effect by facilitating the stability of m6A-modified miR-30d in pancreatic ductal adenocarcinoma (PDAC) [136]. Mechanistically, DF2 directly binds to PPaRα, mediating its mRNA stability to regulate lipid metabolism [137]. These evidences indicate that YTH family proteins play an important role in hypoxia, glycolysis, and lipid metabolism.

### Tumor immunity

Innate immune response and receptive immune response are two major immune responses in human body. Dendritic cells (DCs), CD8^+^ T cells and other immune cells play an important role in receiving immune response in tumor microenvironment. In DF1-dificient mice, increased immunity capacity and reduced melanoma tumor volume were shown. Han et al. found that DF1 could mediate immune evasion and was sufficient to inhibit tumor growth. Deletion of DF1 specifically in classical dendritic cells (cDCs) decreased lysosomal proteases translation, which further augmented the cross-presentation of tumor neoantigens to CD8^+^ T cells. In addition, the immunotherapy efficiency of PD-L1 blockade was increased in DF1-KO mice [138]. Another study reported that DF2 functioned as a tumor suppressor, improved immunotherapy by decreasing the stability of PD-1, CXCR4, and SOX10 mRNA in the m6A-modified mRNA degradation pathway of melanoma cells [58].

DF2 has the same effect of immune promotion in intrahepatic cholangiocarcinoma (ICC). DF2 directly recognized the m6A 3′UTR region of PD-L1 mRNA and promotes its degradation in an m6A-dependent manner [139]. DF2 expression enhanced NK cells proliferation, homeostasis, IL-15-mediated survival, and antitumor ability. The novel positive feedback loop between STAT5 and DF2 as well as the IL-15 leading to NK cell function [140]. In lung cancer, DC1 is positively involved in CD8^+^ T cell infiltration and PD-L1 therapy by assisting in regulating m6A-modified circIGF2BP3 circularization. CircIGF2BP3 improved PD-L1 activity abundant through circIGF2BP3/PXP3/PD-L1 axis, therefore elevates immune escape of CD8^+^ T cells [141]. So far, evidences suggest that member of the m6A methylation modulators-YTH family may enhance tumor immunity and inhibit immune cell infiltration in different cancer.

### Anticancer therapy

So far, there is no specific synthesis inhibitor for members of the YTH family, while several drugs have been found to alleviate the tumor-promoting function of YTH domain-containing proteins. As mentioned above, DF1 and DF3 jointly promote m6A-modified YAP translation. And this study also found that the decreased m6A sites of YAP mRNA were correlated with enhanced tumor drug sensitivity of cis-cisplatin complexes (DDP) [104]. These evidences provide a new perspective on whether the YTH family proteins affect the drug resistance or sensitivity of tumor cells to DDP. In liver cancer, METTL3 depletion enhanced sorafenib resistance via METTL3/FOXO3 axis. It was found that FOXO3 plays an essential role in mediating the m6A-dependent chemo-sensitivity of HCC via inhibiting the autophagy signaling pathway. DF1 significantly stabilizes the expression level of FOXO3 in the m6A-dependent manner [142]. Therefore, DF1 may as promising novel therapeutic targets for overcoming sorafenib resistance in HCC. In LUAD, ammonium tetrathiomolybdate (ATTM) showed the tight association of DF1. Knockdown of DF1 partially blocks the ATTM-induced lung cancer cell growth [143]. Therefore, the therapeutic efficiency ATTM may be positively correlated with DF1 expression level. With the development of immune research, some studies have found that YTH family proteins have a certain relationship with PD-L1 blocking immunotherapy.

As mentioned above, the deletion of DF1 leads to the enhanced therapeutic efficacy of PD-L1 checkpoint blockade, which may indicate that DF1 is a potential treatment in antitumor immunity [138]. The exact mechanism of the DF2/PD-1 axis has previously been demonstrated [105]. DF2 may act as an antitumor immune target via decreasing PD-1 abundance.

## Conclusion

Among the hundreds of post-transcriptional modification types of cellular RNA, m6A is the most prevalent epigenetic modification. In recent years, it has gained rapid advantage in the field of RNA modification. M6A RNA modification and its corresponding binding proteins are emerging as critical regulators of RNA transcription, pre-mRNA splicing, mRNA stability, exporting, translation, tumorigenesis, metastasis, miosis, and immunity. Multiple examples showed various capabilities of m6A readers and further discovered the cross-talk and competition between different m6A readers. For example, DF1, DF2, and DF3 showed a unified model for regulating m6A-modified mRNA degradation and cell differentiation [60]. In this review, the basic structure, physiological function, and research progress of m6A readers–YTH domain-containing proteins in human cancers are summarized. However, a complete understanding of the m6A RNA domain is still far off. First, plenty of unknown fields still exist about the exact and fully biological functions of YTH domain-containing proteins in the region of m6A RNA. Second, though the underlying mechanism of the relationship between m6A readers and human cancers is significantly advanced, challenges about comprehensive exploration in this field still exist. The correlation between m6A readers, as well as the interaction between m6A writer–eraser-readers, and the tumorigenesis and metastasis of human cancers still lack understanding. In clinical therapeutic practice, the potential biomarkers for tumor diagnosis and prognosis still need to be further explored. All these vague points need further investigation.

In conclusion, the development of technology and in-depth research have made us realize the significance of m6A domain-containing proteins. Further researches on the molecular mechanisms of m6A methylation and its regulatory factors can shed light on the clinical diagnosis and targeted therapy of tumors.

## Data Availability

All data relevant to the review are available upon reasonable request.
